# Identification of co-expression gene networks, regulatory genes and pathways for obesity based on adipose tissue RNA Sequencing in a porcine model

**DOI:** 10.1186/1755-8794-7-57

**Published:** 2014-09-30

**Authors:** Lisette J A Kogelman, Susanna Cirera, Daria V Zhernakova, Merete Fredholm, Lude Franke, Haja N Kadarmideen

**Affiliations:** 1 Department of Veterinary Clinical and Animal Sciences, Faculty of Health and Medical Sciences, University of Copenhagen, Grønnegårdsvej 7, 1870, Frederiksberg, Denmark; 2 Department of Genetics, University Medical Centre Groningen, P.O. Box 30001, 9700 RB Groningen, The Netherlands

**Keywords:** Obesity, RNA sequencing, Gene co-expression networks, Regulatory genes, Systems biology, Osteoporosis

## Abstract

**Background:**

Obesity is a complex metabolic condition in strong association with various diseases, like type 2 diabetes, resulting in major public health and economic implications. Obesity is the result of environmental and genetic factors and their interactions, including genome-wide genetic interactions. Identification of co-expressed and regulatory genes in RNA extracted from relevant tissues representing lean and obese individuals provides an entry point for the identification of genes and pathways of importance to the development of obesity. The pig, an omnivorous animal, is an excellent model for human obesity, offering the possibility to study in-depth organ-level transcriptomic regulations of obesity, unfeasible in humans. Our aim was to reveal adipose tissue co-expression networks, pathways and transcriptional regulations of obesity using RNA Sequencing based systems biology approaches in a porcine model.

**Methods:**

We selected 36 animals for RNA Sequencing from a previously created F2 pig population representing three extreme groups based on their predicted genetic risks for obesity. We applied Weighted Gene Co-expression Network Analysis (WGCNA) to detect clusters of highly co-expressed genes (modules). Additionally, regulator genes were detected using Lemon-Tree algorithms.

**Results:**

WGCNA revealed five modules which were strongly correlated with at least one obesity-related phenotype (correlations ranging from -0.54 to 0.72, P < 0.001). Functional annotation identified pathways enlightening the association between obesity and other diseases, like osteoporosis (*osteoclast differentiation*, P = 1.4E^-7^), and immune-related complications (e.g. *Natural killer cell mediated cytotoxity*, P = 3.8E^-5^; *B cell receptor signaling pathway*, P = 7.2E^-5^). Lemon-Tree identified three potential regulator genes, using confident scores, for the WGCNA module which was associated with *osteoclast differentiation*: *CCR1, MSR1 and SI1* (probability scores respectively 95.30, 62.28, and 34.58). Moreover, detection of differentially connected genes identified various genes previously identified to be associated with obesity in humans and rodents, e.g. *CSF1R* and *MARC2*.

**Conclusions:**

To our knowledge, this is the first study to apply systems biology approaches using porcine adipose tissue RNA-Sequencing data in a genetically characterized porcine model for obesity. We revealed complex networks, pathways, candidate and regulatory genes related to obesity, confirming the complexity of obesity and its association with immune-related disorders and osteoporosis.

## Background

Obesity is a complex common health problem, and because of its exponential growth in prevalence in the last decades, the World Health Organization (WHO) has recognized it as a global epidemic since 1997. Obesity is an excess of adipose tissue, resulting from an imbalance between energy intake and energy expenditure [1]. As adipose tissue plays a key role in the control of energy balance by secreting e.g. hormones, cytokines and growth factors, a disturbance in this tissue is strongly associated with other severe diseases such as type 2 diabetes, cardiovascular diseases and various types of cancer [2].

In this study, we use subcutaneous adipose tissue from a previously established porcine model [3] for studying the genetics of obesity. The value of the pig as a model for obesity has been investigated and proven by diverse studies, as the pig (omnivorous like humans) shares metabolic, digestive and cardiovascular features with humans [4,5]. This was also obvious from a previous study of our F2 pig population which showed a strong genetic divergence for diverse obesity-related traits extensively phenotyped [3,6].

Obesity is highly heritable, with heritability estimates between 40 and 70 percent [7], and although many studies have focused on finding loci involved in obesity (e.g. using genome-wide association studies (GWAS) [8]), the genetic risk variants identified to date explain a limited proportion of the heritability. In addition to genetic approaches, transcriptomic analyses have shown to be useful in studying complex diseases. Transcriptomic analyses give insight into the intermediate step between genes and its function, providing the opportunity to better understanding the biological mechanisms [9]. Microarray technologies have been the main platform in recent years [10] and microarray expression data gave the opportunity of both assessing thousands of genes at the same time while providing a better understanding of the underlying biological processes of many complex diseases [11-13]. Expression data are extensively used to detect differentially expressed genes, but network approaches have also gained ground to reveal more of the complex transcriptional regulation by detecting sets of highly co-regulated genes. Clusters of co-regulated genes that share a common function, called ‘modules’, are thought to work together in a network and correspond to, for example, biological pathways. Several network approaches are available, such as the Weighted Gene Co-expression Network Analysis (WGCNA) method (based on correlation patterns between expression profiles) [14] which has proven its superiority over Partial Correlation and Information Theory (PCIT) methods [15], and the Lemon-Tree project (based on probabilistic graphical models) [16]. We have previously applied WGCNA approaches to complex traits in animal populations [17,18], and the method has also proved to be reliable in various human diseases, e.g. different types of cancer [19,20].

A relatively novel platform for gene expression data is RNA Sequencing (RNA-Seq), which allows us to study the complete transcriptome in more detail and with more precise measurements in comparison with microarray platforms, also facilitating the discovery of novel genes [21]. This has already been proven by Iancu et al. [22] who compared the WGCNA approach applied to microarray expression data and RNA-Seq data. Their results showed that the greater sensitivity and dynamic range results in a better estimation of network properties, such as network density and centralization. The huge advantage of detecting novel genes for complex traits will also be a major opportunity in network approaches, where the biological information included in the network helps in revealing the function of the novel genes. To date, a limited number of studies have analyzed RNA-Seq data using the WGCNA approach to investigate complex diseases and traits, e.g. psoriasis [23], heart failure in a mouse model [24] and alcohol use disorders in a mouse model [25]. Moreover, the WGCNA approach has also been used on RNA-Seq data of human subcutaneous adipose tissue, revealing a cluster of genes associated with serum triglyceride regulation [26]. Also the Lemon-Tree algorithms, previously published as LeMoNe, have showed their value in different biological traits [27-29]. The combined use of high-throughput omics data from clearly characterized groups of individuals for complex diseases and mathematical models to build gene co-expression and regulatory networks is at the core of systems biology methods [13,17,30].

In the present study we applied the WGCNA method and Lemon-Tree algorithms, using RNA-Seq data, with the aim to elucidate transcriptomic regulations of obesity by detecting pathways, novel and regulator genes involved in its pathogenesis. To our knowledge, this is the first study using systems biology and network approaches to study the overall complex transcriptional regulation of obesity using RNA-Seq data in a genetically characterized porcine model.

## Results and discussion

### The genetic background of selected animals

We previously created and published one aggregate genotypic value (or predicted genetic risks) called “the Obesity Index” (OI), representing the degree of obesity per pig from a larger population of 279 pigs resulting from an F2 intercross between Duroc and Göttingen Minipigs [6]. The normal distribution of OI among the entire population clearly showed the existence of large genetic variation between animals. Based on the distribution of the OI we categorized animals into three extreme groups, one for obesity, one for leanness and a third group from the middle of the OI distribution. Subcutaneous adipose tissue was collected for paired-end RNA-Seq from 12 lean (L), 12 intermediate (I) and 12 obese animals (O) totaling 36 samples. Descriptive statistics of the OI and a selection of other obesity-related phenotypes within the three subgroups of pigs are presented in Table 1. Among the selected animals, there is a large difference in age at slaughter (L: 309 days, I: 234 days, O: 218 days), as they were slaughtered at approximately 100 kg. In addition, other obesity-related phenotypic measurements showed a significant difference between the three groups, as shown in Table 1.

**Table 1:** Descriptive statistics (mean and standard deviation) and test of difference of means for a selection of obesity-related traits for the three subgroups

	**Gender**^**1**^	OI***	Wgt***	AbdCirc***	ADG***	**DXA**_fat_**	**SL**_fat_**	**SL**_%meat_**
Lean	7/5	-2.47 (0.75)	80.13 (14.98)	112.09 (12.02)	0.29 (0.03)	1833.35 (660.30)	1.70 (1.37)	48.12 (6.88)
Intermediate	6/6	0.07 (0.11)	93.71 (16.87)	122.83 (7.85)	0.45 (0.04)	2031.00 (512.69)	2.68 (0.87)	43.29 (3.70)
Obese	6/6	2.4 (0.36)	113.75 (11.97)	134.25 (7.58)	0.59 (0.05)	3050.22 (907.17)	3.26 (1.17)	41.4 (7.44)

^1^Presented as frequency male/female.

***Highly significant, P-value < 0.001.

**Significant, P-value < 0.01.

Wgt: weight at appr 7 months of age, AbdCirc: abdominal circumference at approximately 7 months of age, ADG: average daily gain from birth to slaughter, SLfat: weight of leaf fat at slaughter, SL%meat: percentage of meat content at slaughter.

### Weighted Gene Co-expression Network Analysis (WGCNA)

**Figure 1:**
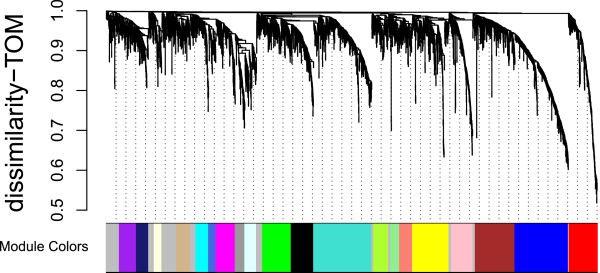
Gene dendrogram showing the co-expression modules defined by the WGCNA labeled by colors.

We applied the WGCNA approach using the count data resulting from RNA-Seq of 36 porcine subcutaneous adipose tissue samples. The WGCNA analysis relies on the assumption that strongly correlated expression levels of a group of genes indicate that those genes work cooperatively in related pathways, contributing to the resulting phenotype. In addition, genes may also cluster together as a result of a common set of transcription factors. The co-expression network was constructed using 3,532 selected genes and clusters of highly co-expressed genes (modules) were detected and assigned to module colors (Figure 1). In total, we identified 20 modules, labeled by colors, with each containing at least 50 genes.

**Figure 2:**
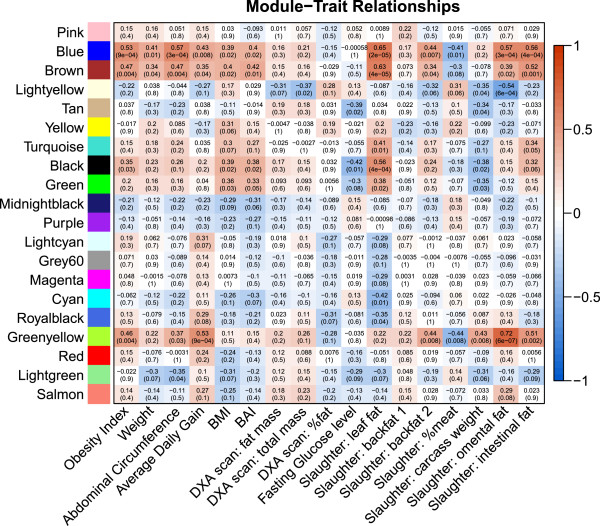
**Matrix with the Module-Trait Relationships (MTRs) and corresponding p-values between the detected modules on the y-axis and selected obesity and obesity-related traits on the x-axis.** The MTRs are colored based on their correlation: red is a strong positive correlation, while blue is a strong negative correlation. Explanation of the traits: Weight, Abdominal Circumference, BMI (Body Mass Index: weight/length^2^), and BAI (Body Adiposity Index: abdominal circumference/length^1.5^) were measured at approximately 7 months of age. DXA scanning was performed at approximately 2 months of age. Fasting glucose levels were determined using an oral glucose tolerance test at approximately 7 months of age. Animals were slaughtered at approximately 100 kg (~7 months). More detailed information can be found in Kogelman et al., 2013 [3].

For each module an eigengene was calculated that explained between 33 and 72 percent of the expression variation. The Module-Trait Relationships (MTRs) with all selected obesity and obesity-related (OOR) traits were calculated by correlating the module’s eigengene to the traits of interest (Figure 2) and used for selection of modules for downstream analysis. The matrix representing all MTRs shows that several modules are highly correlated with one or more OOR trait, and it also shows a clear distinction in the MTR of modules between the different OOR traits. Modules were selected when they had a MTR > 0.5 with at least one OOR trait and genes in the modules were retained in the module based on their intra-modular connectivity. On the basis of those selection criteria we selected five modules for functional annotation: the Blue Module (275 genes), the Brown Module (62 genes), the Lightyellow Module (21 genes), the Black Module (105 genes) and the Greenyellow Module (41 genes). All genes present in those modules are presented in Additional file 1.

### Functional enrichment of modules

After correcting for potential gene length bias (see Methods), as longer and highly expressed genes have a greater chance of being detected [31], we identified overrepresented Gene Ontology (GO) terms and KEGG pathways in the selected modules using GOSeq. P-values were adjusted using the Benjamini-Hochberg (BH) correction. The most significant GO terms and KEGG pathways are presented in Table 2.

**Table 2:** Overview of the most significantly overrepresented KEGG pathways and GO terms associated with the modules detected using WGCNA

**WGCNA module**	**KEGG pathway or GO-term**	**Frequency in module**	**Number of genes in term**	**P**_**adj**_
**Blue**	KEGG: Osteoclast differentiation	12	52	1.40E-07
	KEGG: Natural Killer cel mediated cytotoxity	8	34	3.76E-05
	KEGG: B cell receptor signaling pathway	7	29	7.24E-05
	GO: Immune system response	39	516	5.57E-11
	GO: Cell activation	26	217	2.53E-10
	GO: Regulation of immune system response	28	82	1.22E-09
**Black**	GO: Extracellular region	40	382	5.53E-06
	GO: Extracellular matrix	21	121	3.61E-05
	GO: Proteinaceous extracellular matrix	19	97	3.61E-05
**Lightyellow**	GO: Nucleolus	8	124	1.30E-02
	GO: Ribosome biogenesis	4	18	3.40E-02
	GO: Nuclear par	12	437	3.40E-02

**Figure 3:**
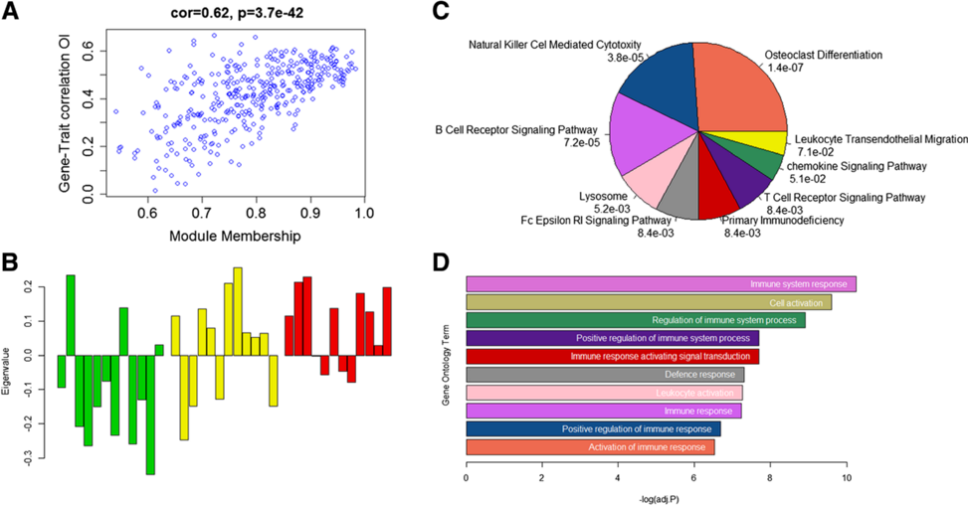
**WGCNA module (Blue) associated with immunity. A)** Association between the Module Membership and Gene-Trait correlation within the blue module. **B)** Module eigengene values (y-axis) across samples (x-axis), with the 12 lean animals colored green, the 12 intermediate animals colored yellow and the 12 obese animals colored red. **C)** Pie chart of all significant KEGG pathways in the blue module. **D)** The top 10 significant Gene Ontology terms in the Blue module.

The Blue module (eigengene), having the highest correlation with the OI (MTR_OI_ = 0.53), showed an overrepresentation of immune-related GO terms and KEGG pathways. As the Blue module is a rather large module, we looked into some intra-modular characteristics: the intra-modular connectivity and the Gene-Trait correlations with the OI (Figure 3A). This plot evidently showed that genes with a high module membership were also highly correlated with OI, motivating us to reduce the module based on the module membership. Here we increased the Module Membership threshold to 0.9, resulting in 69 genes. The expression profile of the eigengene of the Blue module showed a clear under-expression for the lean animals, while it showed overexpression in the obese individuals (Figure 3B).

These expression profiles are as expected, as various studies have shown that an increase of adipose tissue is correlated with an increase of several types of immune cells, particularly with macrophages [32,33] which are key players in the initiation of a chronic inflammatory state in obesity [34]. As expected for the 69 genes, the significant GO terms and KEGG pathways were still nearly all immune related, except that the most significant KEGG pathway was *osteoclast differentiation* (P_adj_ = 1.4E^-7^) (Figure 3C & 3D). Osteoclasts are derived from macrophages, one of the most up-regulated immune cells in adipose tissue of obese individuals, and are therefore also closely linked to many immune diseases [35]. Bone marrow houses two kinds of stem cells: the mesenchymal stromal cells which are precursors for osteoblasts and adipocytes and the hematologic stem cells originating from osteoclasts. Moreover, there is an important communication between adipose tissue and skeleton where factors secreted by adipocytes affect bone remodeling, i.e. leptin, adiponectin, pro-inflammatory cytokines as Interleukin 6 (IL-6) [36,37]. IL-6 is known to be an important regulator of the immune and hematopoietic systems and it has been associated with osteoporosis disease and rheumatoid arthritis [38,39]. Osteoporosis is a polygenic trait [40], whereby increased bone fragility results from increased adipocytes and osteoclastogenesis and insufficient osteoblastogenesis [41]. When looking at the functions of the different genes present in the Blue module, we find many genes which have a clear function in the immune system and also have been associated with osteoclast differentiation, e.g. *BCL2A1*, *DOK2*, *PTPN6* and several genes encoding cell surface molecules (e.g. *CD45* and *CD68*). Another gene in this module is *SPI1. SPI1* is encoding the transcription factor PU.1 protein which activates gene expression during myeloid and B-lymphoid cell development. A study of Wang et al. [42] has shown that PU.1 is expressed in white adipose tissue and plays a role in adipogenesis. Moreover, variations in *SPI1* play a role in osteoclastogenesis as for example, PU.1 deficient mice develop osteoporosis [43], and it increases the risk of fracture by its effect on *ALOX15*[44].

Other highly significant associated pathways in this module are all immune-related, of which the most significant is *Natural killer cell mediated cytotoxity* (P-value = 3.8E^-5^). In fact, obesity causes morphological changes in adipose tissue, resulting in a state of chronic low-grade inflammation [45]. Furthermore, natural killer (NK) cells are critical in the innate immune response, less examined in association with obesity, but it has been shown that diet-induced obese mice show a reduced NK cytotoxity after infection [46]. Another study showed an increased level of NK cells in healthy obese compared with unhealthy obese individuals, suggesting its importance in metabolic processes [47]. Several studies have shown and investigated the link between the immune system and metabolism [48,49], also in combination with obesity [50,51]. This also explains the significant association of the other KEGG pathways and GO terms in this module.

The Black module (MTR_OI_ = 0.35) shows a strong reverse correlation (-0.42) with fasting glucose levels (FGL). The KEGG pathways are not significant after BH correction, but before BH correction the most significant pathway is *ECM-receptor interaction* (P = 0.001). Several GO terms related to this extracellular matrix (ECM) are found to be significantly overrepresented, also after BH correction, e.g., *extracellular region* (P_adj_ = 5.5E^-6^), *extracellular matrix* (P_adj_ = 3.6E^-5^) and *proteinaceous extracellular matrix* (P_adj_ = 3.6E^-5^). As we are interested in the genes which are involved in the pathways representing the high positive correlation with fatness, but with a high negative correlation with glucose levels, we examined the association of the genes between the two traits. We selected leaf fat at slaughter (SL_fat_) and FGL as traits of interest because of their high correlations. The correlations of the expression profiles with these traits show that there is a wide variation in their correlations with both traits, and that there is a weak negative correlation (-0.23) between the Gene-Trait correlations of SL_fat_ and FGL. Next, we only selected genes having a correlation >0.4 with both SL_fat_ and FGL, resulting in a selection of 36 genes, of which 24 were assigned a gene name, for further functional annotation. Of these genes we will only comment on the most relevant in relation to obesity. *ADAMTS-12* is a metalloprotease necessary for normal immunological response [52]. The *PFPK* gene (phosphofructokinase, platelet) is a key regulatory enzyme in glycolysis. In the first GWAS presented on obesity, this gene was found to be associated, but did not get validated in the replication stage [53]. However, differential gene expression in the visceral adipose tissue shows differential expression of the *PFPK* gene in obese vs. lean individuals [54]. Two of the genes in the modules are proprotein convertase subtilisin/kexins (PCSK): the *PCSK5* and *PCSK6* gene. Several studies have shown their relevance in metabolic-associated processes, for example high-density lipoprotein (HDL) metabolism [55,56]. The *BDKRB2* (BK type 2 receptor) gene has been suggested to be acting as a genetic modulator of glucose homeostasis, potentially increasing susceptibility to diseases like diabetes [57]. The Probable G-protein coupled receptor 133 (encoded by the *GPR133* gene) has been associated using GWAS to body weight control in mice [58] and height control in human [59]. The *MFAP5* gene is also present in the Black module, encoding the microfibrillar-associated protein 5. This is an extracellular matrix glycoprotein, shown to be expressed in adipose tissue, and its expression is positively correlated with BMI, and change in body fat mass [60]. Moreover, Vaittinen *et al*. [60] also found correlations between *MAFP5* expression and insulin resistance markers.

The third investigated module is the Lightyellow Module (MTR_OI_ = -0.22), showing a high MTR with omental fat at slaughter (-0.54). This module does not show any significant KEGG pathways after BH correction, but several significant overrepresented GO terms related to transcription were detected, e.g. *nucleolus* (P_adj_ = 0.013), *ribosome biogenesis* (P_adj_ = 0.034), *nuclear part* (P_adj_ = 0.034), *DNA-dependent transcription*, *elongation* (P_adj_ = 0.038). All these processes are involved in gene expression, which is altered under obesity. The most highly interconnected genes in this module are *AMD1*, *NOL9*, *EIF4A1*, *POLR1C* and *ABCE1. AMD1*, the hub-gene in this module, plays a key role in polyamine biosynthesis. Polyamines have shown to affect growth and development of adipose tissue, and increased levels of polyamines have been associated with childhood obesity [61]. Moreover, in an Indian childhood cohort *AMD1* has been associated with obesity and plasma leptin levels, speculating that *AMD1* influences the susceptibility to obesity by modulating the polyamine metabolism or DNA methylation [62]. *NOL9* plays a role in ribosomal RNA (rRNA) processing [63] which takes place in the nucleolus. It has previously been shown that the *EIF4A1* gene plays a role in the protein synthesis pathway which was altered in insulin-resistant obese individuals [64]. Furthermore, *EIF4A1* was negatively correlated with the insulin receptor gene (*InsR*). *POLR1C* is important in RNA polymerization, which is necessary for transcription. *ABCE1* is involved in viral assembly by inhibiting the action of RNase L, a regulator of innate immunity. It has been shown that RNase L plays an important role in adipogenesis regulation [65] and is able to restore insulin response in muscle cells of obese individuals, suggesting a role for RNase L in “healthy obese” subjects [65]. This finding is in agreement with a study of Mahdi et al. [66] that showed a correlation between the expression levels of *ABCE1* and insulin secretion, following the same WGCNA approach as this study.

Neither the Greenyellow module (MTR_OI_ = 0.45) nor the Brown module (MTR_OI_ = 0.47) showed any significant KEGG pathways or GO terms after BH correction.

### Differential network analysis

Previous studies have shown the potential of studying the co-expression patterns of sub-networks, thereby comparing two different states of the phenotype of interest [67]. Therefore, we created and compared the lean sub-network and obese sub-network using the expression data of the 12 low OI animals and the 12 high OI animals. For both sub-networks the gene dendrogram was created and modules were subsequently defined to colors (results not shown). Both sub-networks revealed clearly different modules; however, the obese modules were not preserved in the lean sub-network, and vice versa. Biologically, this shows different active pathways in the lean versus obese animals, as expected.

**Figure 4:**
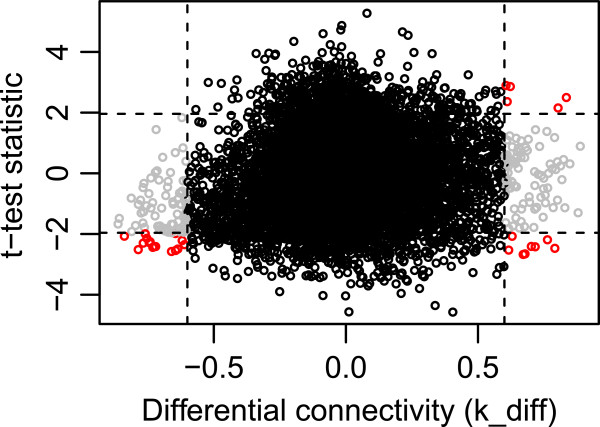
**Plot of the differentially co-expressed genes with on the y-axis the*t*-test statistic (lean vs. obese) and on the x-axis the differential connectivity.** Genes are colored grey when they are differentially connected and colored red if they are also being differentially expressed. In total, 29 genes (19 of which were assigned a gene name) were colored red, which were selected for functional annotation. Discontinuous lines represent thresholds for gene selection: absolute *t*-test statistic > 1.96 and absolute differential connectivity > 0.6.

To indicate which genes are acting differently in the sub-networks and consequently potentially being involved in the obese phenotype, we indicated which genes were differentially co-expressed. Modules are constituted of a group of highly interconnected genes, and as a consequence of scale-free topology assumptions, it will consist of many low interconnected genes and a few highly interconnected genes (hub genes). Those hub genes are believed to be biologically important, as they represent tightly regulated processes. We expect that hub genes that behave differently in a certain condition will have a key role in that particular condition. The differential connectivity (k_diff) represents the change in connectivity between the lean and obese sub-networks. In total, we detected 185 differentially connected genes (absolute differential connectivity > 0.6). To assign these genes to biologically relevant genes, they were further selected based on their difference in expression levels between the lean and obese condition (absolute *T*-test statistic > 1.96). This resulted in the detection of 29 genes, 19 of which were assigned a gene name (Figure 4) and 12 of which were also present in one of the selected modules.

We found eight genes (*IFI35*, *SNX19*, *MARC2*, *MORN2*, *PLA2G16*, *CCL5*, *CACNA2D1*, and *REV1*) that were hub genes in the obese sub-network, but lowly interconnected in the lean sub-network. All except *IFI35* and *REV1* are up-regulated in the obese animals. Many of the obese hub-genes are, as expected, active in obesity-related pathways, where we can see a subdivision in fatness related pathways and immune-related pathways. Both *IFI35* and *CCL5* are immune-related genes. *IFI35* (interferon-induced protein 35) is a cytokine important for communication between cells in the innate immune response. A recent study showed that *IFI35* negatively regulates the RIG-I antiviral signaling [68], which in turn activates the innate immune response. These patterns are in concordance with the down-regulation of *IFI35* in our obese pigs, as it has been accepted that the native immune response is triggered in obese individuals [69]. *CCL5* (chemokine (C-C motif) ligand 5) is secreted by bone marrow stromal cells and encodes a protein which is also known as *RANTES* (regulated on activation, normal T cell expressed and secreted). It plays a key role in recruiting leukocytes during immune response and plays a role in inducing the activation of Natural Killer cells [70]. It may also play a role in the inflammation of obese human white adipose tissue [71], as it shows up-regulation in obese subjects [72]. *SNX19* (sortin nexin 19) encodes a membrane-associated protein complex which is associated with coronary heart disease and myocardial infarction. It can also interact with IA-2 which is a major auto-antigen in type 1 diabetes and a regulator of insulin secretion [73]. Several of the obese hub-genes are also related to fat and body weight. The first one is *MARC2* (Mitochondrial Amidoxime Reducing Component 2), which has been associated with decreased total body fat and increased circulating glucose levels in mice [74]. The *PLA2G16* (adipocyte phospholipase A2 (AdPLA) gene, also present in the Greenyellow module, encodes an enzyme that is mainly found in adipose tissue and catalyzes the release of fatty acids from phospholipids in adipose tissue [75]. Genetic variants of this gene have been associated with neutral lipid storage disease [76] and growth traits in cattle [77]. AdPLA-deficient ob/ob mice show an increased lipolysis, a reduced adipose tissue mass and triglyceride content, but show a normal adipogenesis, showing its role in the control of adipocyte lipolysis and thereby its important role in the development of obesity [78]. The *CACNA2D1* gene (Voltage-dependent calcium channel subunit alpha-2/delta-1) encodes a protein in the voltage-dependent calcium channel complex. In a meta-analysis on body fat in mice it was shown to be associated with body fat percentage [79] and has been associated with carcass and meat quality traits in cattle [80,81]. The exact function of the *MORN2* gene is unknown; this gene was also present in the Greenyellow module. The last obese hub-gene is *REV1*, which encodes a DNA repair protein. The fact that this gene is down-regulated in obese animals may be explained by the fact that the DNA repair process is perturbed under obesity. It has been seen, for example, that mutations in genes encoding for DNA repair proteins are associated with developmental inhibition, immunodeficiency and increased risk of cancer in humans [82].

In addition, we found 11 genes (*CSF1R*, *EVI2B*, *SAMHD1*, *PCD1A*, *CD68*, *FAM105A*, *P2Y12R*, *NCEH1*, *SPI1*, *FRMPD4* and *PigE-108A11.6*) which were hub-genes in the lean network but weakly interconnected in the obese sub-network. All these genes are up-regulated in the obese animals. All of them, except *FRMPD4*, were also found in one of the WGCNA modules. Except for the *SAMHD1*gene, they were all found in the Blue module; *SAMDHD1* was present in the Black module. Several of the 11 genes are immune-related, e.g. *CSF1R*, *SAMHD1*, *PCD1A*, *CD68*, *SPI1* and *PigE*-*108A11.6. CSF1R* (Colony stimulating factor 1 receptor) controls the production, differentiation and function of macrophages [83] and its expression has been correlated with body fat percentage in humans [84]. *SAMHD1* (SAM domain and HD domain-containing protein 1) is an enzyme exhibiting phosphohydrolase activity, converting nucleotide triphosphates to a nucleoside and triphosphate. *PCD1A* is the porcine cd1 antigen involved in the presentation of lipid antigens to T cells, but its precise function is unknown. *CD68* encodes a glycoprotein that binds to low-density lipoprotein, expressed on monocytes/macrophages. Its expression was significantly up-regulated in acquired obesity in monozygotic twin pairs and correlated to liver fat and insulin resistance [85]. *SPI1* has been found in the Blue module in WGCNA, and its relation with adipogenesis and ostoclastogenesis has been discussed. *PigE-108A11.6* is the orthologous of *LILRB5* (leukocyte immunoglobulin-like receptor subfamily B member 5), which is expressed in immune cells where they bind to MHC class 1 molecules on antigen-presenting cells and inhibit stimulation of an immune response. It has been found to be up-regulated in omental adipose tissue in obese individuals [86]. Also various other genes were detected among the hub-genes in the lean animals. The *P2Y12R* gene encodes a protein which is an important regulator in blood clotting and consequently associated with heart attacks [87]. Moreover, insulin inhibits blood platelet aggregation by suppressing the P2Y12 pathway, and therefore type 2 diabetes leads to up-regulation of the P2Y12 pathway, resulting in increased platelet reactivity [88]. The *NCEH1* (Neutral cholesterol ester hydrolase 1) gene encodes an enzyme which is located in the endoplasmic reticulum which plays a role in the regulation of the levels of platelet activating factor and lysophospholipids. *NCEH1* has a key role in the reverse cholesterol transport in macrophages, thereby playing a critical role in human atherosclerosis [89].

### Detection of regulator genes

Several of the detected modules showed an overrepresentation of obesity-related KEGG pathways or GO terms, but the WGCNA approach did not reveal potential gene regulators of detected modules, as WGCNA is an undirected network. The challenge of finding potential regulators of significant gene modules related to obesity was addressed by using Lemon-Tree algorithms (available at https://code.google.com/p/lemon-tree/). Lemon-Tree created a set of potential regulators consisting of transcription factors and signal transducers using the GO categories ‘transcription factor activity’ and ‘signal transducer activity. Those potential regulators were then assigned to nodes with corresponding probabilistic scores for being a “regulator”. Genes will have a high probabilistic score in case there is a differential expression pattern on each side of the particular tree node. After generation of multiple statistically equal partitions of conditions for each cluster of genes, the algorithm uses an ensemble approach to sum the strength with which a regulator participates in each regulatory tree. The overall statistical confidence was calculated and used for prioritizing regulators, represented by the global probabilistic score (Prob. score). Mathematical details of the Lemon-Tree algorithms can be found in Joshi et al. [16]. In this way, we built a regulatory module network using the RNA-Seq expression data and assigned high-scoring regulator genes to the detected clusters of co-expressed genes. We detected in total 43 tight clusters of no less than 10 genes, which resulted in a total of 1417 genes in the clusters. A pre-defined group of genes was tested to see whether they may regulate the expression levels of the genes in a particular module, represented by the global probabilistic score (Prob. Score). We tested the significance of the assigned regulators using a *t*-test, showing that the assigned regulators are significantly different from randomly assigned regulators (P-value = 0.000).

**Figure 5:**
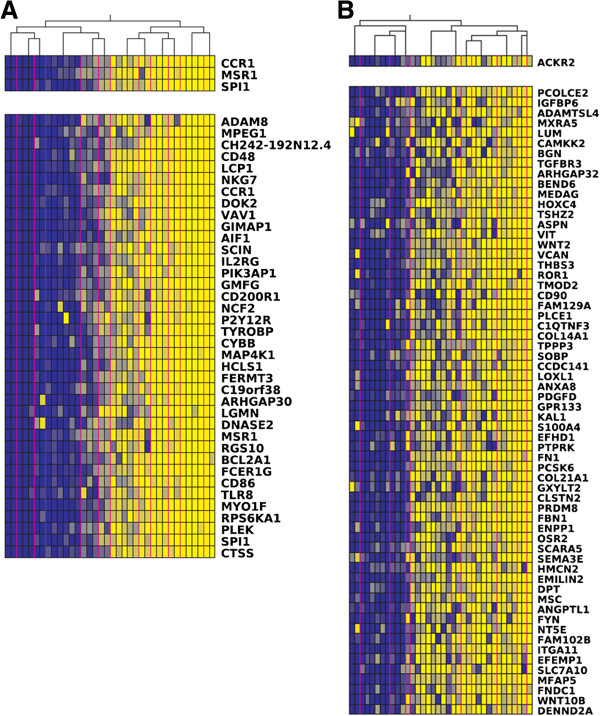
**Color coded expression values of two clusters resulting from the Lemon-Tree algorithm. A)** Cluster1, and **B)** Cluster10. The upper panel represents the high-scoring regulator genes, ordered by their probabilistic score (highest on top). The lower panel represents the genes present in the cluster. Each column represents a sample. The expression of the genes and regulator gene is color coded, with dark blue representing low expression, while bright yellow indicates highly expressed genes. The hierarchical tree on top of the figure is the tree used to assign the regulator gene. The vertical pink colored line represents the partition of samples defined by the first node of the tree.

As the Blue module resulting from the WGCNA showed a strong association between obesity, immunity and osteoporosis, we investigated the regulator genes of this module. As expected, many of the genes in the Blue module were present in one cluster (cluster1) using Lemon-Tree algorithms. Secondly, we assigned the regulators to the clusters using transcription factors and signal transducers as potential regulators. Associations between these potential regulator genes and detected clusters were calculated, and the top 1% of the regulators were selected as high-scoring candidate regulators (40 regulator genes). Three of these regulators were high-scoring regulators in cluster1, and their expression levels were positively correlated with the expression levels of the genes in the cluster: *CCR1* (Prob. score = 95.30), *MSR1* (Prob. score = 62.28) and *SPI1* (Prob. Score = 34.58) (Figure 5A). Both *CCR1* and *SPI1* were also present in the Blue module, where *SPI1* had already shown its relevance to osteoclast differentiation. Statistical scores show that *CCR1* is detected as regulator gene (highest global probability score), encoding the C-C chemokine receptor type 1 protein, which is interacting with the previously mentioned *CCL5* (*RANTES*). It has been mentioned that chemokines play an important role in cell-signaling during immune response. In addition, it has been shown before that *CCR1* is functionally active in human adipose tissue derived stromal cells [90] and plays a role in bone remodeling. Ccr1-deficient mice show a decreased bone mineral density, reduced number of osteoblasts and osteoclast, resulting in an impaired bone formation [91]. Also macrophage scavenger receptors, encoded by *MSR1* (also called *SR-A*), have been associated with osteoclast differentiation, as for example *SR-A* deficient mice show an impaired differentiation of osteoclasts. It was thus concluded that *SR-A* promotes osteoclastogenesis by increasing expression levels of receptor activator of NF-κB (RANK) and related molecules [92]. Again, those results show that there is a link between obesity, the immune system and bone remodeling (osteoporosis) [93,94], and point to a regulatory role in these processes for *CCR1*, *MSR1* and *SPI1*.

We also examined the genes present in the Black module in the Lemon-Tree results. Many of the genes in the Black module clustered together in cluster10. The Lemon-Tree algorithms point to *ACKR2* as regulator gene (Figure 5B), again encoding a chemokine receptor, but in this case its exact function is not known.

## Conclusions

Examining the obesity-related measurements of the 36 RNA-sequenced animals (divided among three genetically extreme groups) showed that there are clear differences between the three groups. Further investigation of their expression profiles using systems biology and network approaches revealed pathways that potentially play a key role in the development of obesity and obesity-related diseases (e.g. inflammation and osteoporosis). Specifically, our network approach WGCNA revealed several clusters of highly interconnected genes of which some were associated to immune-related pathways, which have previously been implicated in the pathogenesis of obesity. A subset of these pathways was also related to *osteoclast differentiation* (P_adj_ = 1.4E^-7^), which has been associated with the immune system and osteoporosis. The co-expression of immune-related genes and their association with osteoclast differentiation shows the close association between obesity, the immune system and bone remodeling diseases like osteoporosis – these results have important implications for obese human patients. Furthermore, Lemon-Tree algorithms detected three regulator genes: *CCR1*, *MSR1* and *SPI1*. Those were previously associated with the immune system and/or osteoclast differentiation, but here we show that those genes may have a key role in the link between obesity and osteoporosis. Another module contained genes previously associated with lipid metabolism and glucose metabolism, which reveals the known close association between obesity and other metabolic diseases like type 2 diabetes. Differential network analysis revealed several genes which showed a different level of activity in the pathways they represented. Many of those genes were previously associated with obesity or the immune system, but not detected using single-gene association studies. This stresses the potential of using systems biology or network biology approaches on complex diseases in order to detect genes which may be important in disease development, and consequently could be potential drug markers.

In conclusion, this study shows the advantage of using systems or network biology approaches in complex diseases to unravel their genetic architecture and transcriptional regulation. We revealed several new pathways and, more importantly, key regulator genes present in those pathways which give a better understanding of the complex transcriptional regulation of obesity. Furthermore, we have confirmed, using our genetically characterized omnivorous porcine model, the known association of obesity with other bone-related and immunity-related diseases at the transcriptomic level, and revealed several genes actively present in those associations.

## Methods

### The F2 pig population and subcutaneous adipose tissue samples

The samples used in this study were derived from a pig resource population established with the purpose of identifying the molecular background for obesity and obesity related traits. For a detailed description of the population and the phenotypes see (Kogelman et al.) [3]. In this study we have only used the Duroc *Göttingen Minipig intercross that comprise a total of 279 animals generated by intercrossing Göttingen minipig boars (Ellegaard A/S) and Duroc sows (DanBred breeding herd). The Göttingen minipig breed is characterized for their small size, and its genetic predisposition for obesity, and for sharing several metabolic impairments seen in humans [95]. The Duroc pig is a production breed, intensively selected for leanness and growth during the last 60 years. This population has been extensively phenotyped for OOR traits, e.g. weight, conformation, dual energy x-ray absorptiometry (DXA) scanning and slaughter measurements. The F2 pigs were produced at the research farm, University of Copenhagen Tåstrup, Denmark. Animal care and maintenance have been conducted according to the Danish “Animal Maintenance Act” (Act 432 dated 09/06/2004) and biological samples were collected according to the Danish “Veterinary Procedures Act” (Act 433 dated 09/06/2004). After slaughtering, a biobank of tissues was created by sampling several tissues for each animal, including subcutaneous adipose tissue. Following, we created one aggregate predicted genetic value based on the selection index theory [96] to represent the degree of obesity in each animal: the Obesity Index (OI) [6]. The OI followed a normal distribution, and based on this distribution animals were categorized in two extreme groups: obese and lean. A third group was categorized as being around the mean of the distribution. We selected 36 animals from those three groups for RNA-sequencing of subcutaneous adipose tissue: 12 low OI (lean), 12 intermediate OI, and 12 high OI (obese). For animal selection, the family structure and the genders of the animals was taken into account, by maximizing the number of directly related animals (full sibs) to three per group and taking care of equal gender distribution within the groups.

### Phenotypic characterization of the selected animals

The phenotypic measurements of the animals were investigated to compare the phenotypic distributions between the three selected groups. Data that were not normally distributed was transformed to approach normality. Differences in means were tested using an analysis of variance model using the R statistical package [97]. All phenotypic measurements were corrected for gender, and in case of a significant effect corrected for batch and age effect, as described before in Kogelman et al. [3]. Results were noted as highly significant with a P-value below 0.001, significant with a P-value below 0.01 and tend to be significant with a P-value below 0.05.

### RNA isolation and RNA-Sequencing

Total RNA was isolated from porcine subcutaneous adipose tissue using the RNeasy lipid Mini kit (Qiagen, Germany) following manufacturer’s recommendations. Briefly, 100 mg of tissue were homogenized in Qiazol buffer using the gentleMACS™ Octo Dissociator system (Milteny Biotec, GmbH, Germany) with M tubes (Milteny Biotec, GmbH, Germany) and the RNA_02 program recommended by the manufacturer. The RNA was DNase treated in order to degrade the remaining genomic DNA. At the end of the protocol the RNA was eluated in 30 μl of RNAse free water. Quantity and quality were assessed by Nanodrop ND-1000 spectrophotometer. Furthermore integrity of the isolated RNA was inspected by electrophoresis in a 1.4% agarose gel and by measuring the RQI value on an Experion™ system (BioRad) using Eukaryote Total RNA StdSens kit (BioRad). The RQI average for all the 36 extracted samples was 8.56.

For the RNA-seq, libraries were constructed using 400 ng of total RNA and TruSeq RNA Sample Prep (Illumina) with Poly-A pull down rRNA depletion following manufacturer’s recommendations. Samples were sequenced on the HiSeq2500 platform, dividing the 36 samples over 4 lanes, using a read length of 100 bp paired-end reads [98]. Before alignment, reads with a low quality and adapters were detected using FastQC and removed. Remaining reads were mapped to the SScrofa10.2.72 genome using default parameters in STAR aligner. This resulted in an average of 30,557,234 uniquely mapped reads per sample, of which on average 81.60 percent was mapped in the intragenic region (within introns or exons). On average 20,390 transcripts were detected among the mapped reads. Read counts were estimated at gene-level using HTSeq [99].

### RNA-Sequencing data normalization and gene selection

Previous studies have been shown that genes with extreme low expression levels are less reliable [100]. Therefore, genes with expression levels equal or lower than 5 were removed from the dataset, resulting in 12,253 genes per sample. The between-sample bias was removed by estimating the library size factor using the estimateSizeFactor() function in DESeq [101]. Normalization was then performed using the voom() variance-stabilization function in the R-package Limma [102], whereby samples were corrected for gender.

### Weighted Gene Co-expression Network Analysis

Because of computational limitations the dataset had to be further reduced for network construction. As it is believed that non-changing genes provide limited information in a co-expression network setting, genes were selected based on their variation (SD > 0.25), resulting in 8,745 genes. Furthermore, based on the connectivity (sum of connection strengths with all other genes) genes were selected for the lean, intermediate and obese sub dataset, as genes with a high connectivity (hub genes) are thought to be more biologically important [103]. The 1500 highest connected genes in each of the lean, intermediate and obese sub-dataset resulted in a joint dataset of 3532 unique genes. Consequently, we built an unsigned co-expression network using the WGCNA R-package [104], using 3532 genes. The adjacency matrix was created by calculating the Pearson’s correlations between all genes, and raised to a power β of 7. The power β was chosen based on the scale-free topology criterion [105], resulting in a scale-free topology index (R^2^) of 0.92. Next, the Topological Overlap Measure (TOM), representing the overlap in shared neighbors, was calculated using the adjacency matrix. The dissimilarity TOM was used as input for the dendrogram, and modules (clusters of highly interconnected genes) were detected as branches of the dendrogram using the DynamicTreeCut algorithm [106]. All modules were assigned to a color. The module eigengene was used to represent each module, which was calculated by the first principal component, thereby capturing the maximal amount of variation of the module. Using the module eigengene, the Module-Trait relationships were estimated by calculating the Pearson’s correlations between the module eigengene and the traits of interest. Those Module-Trait relationships were used to select potential biologically interesting modules for downstream analysis. Modules were selected when they had a correlation >0.5 with at least one of the selected traits. Genes in the module were selected when their intra-modular connectivity with that particular module was >0.6, the intra-modular connectivity with all other modules <0.6. The intra-modular connectivity is calculated as the correlation between the gene’s expression profile and the expression profile of the module eigengene. Another gene characteristic is the Gene Trait correlation: the correlation between the gene’s expression profile and the phenotype of interest.

### Functional annotation

After selection of modules and genes retained in those modules, we investigated those modules using several different module characteristics. An important intra-modular analysis was detection of the correlation between the Module Membership and the Gene-Trait relationships. The Module Membership is based on measuring the intra-modular connectivity: the correlation of a particular gene in the module with the module eigengene. The Gene-Trait relationship is the correlation between the expression profiles of a particular gene with the phenotype of interest. We selected a subset of the modules based on functional annotation. Using BioMart [107] the associated gene names were detected. Gene length bias has been shown to be an important bias in RNA-Seq, as longer genes will be sequenced deeper than shorter genes [31]. Therefore, we used GOSeq [108] to detect overrepresented gene ontology (GO) terms and KEGG pathways, which is able to correct for gene length bias. The Probability Weighting Fuction (PWF) is obtained depending on the gene length, which is used in the Wallenius approximation to calculate overexpressed GO terms and KEGG pathways among the selected modules. To correct for the multiple testing problem, the p-values are adjusted using the Benjamini-Hochberg (BH) correction. GO terms and KEGG pathways were thought to be significant when the adjusted p-value was below 0.05.

### Differential connectivity

The differential connectivity is a measure of the differences in gene interactions between the lean and obese animals, potentially identifying genes which underlie a difference in transcriptional regulation [67]. Therefore, two sub-networks were created: one with only the 12 lean animals (lean sub-network) and one with only the 12 obese animals (obese sub-network). For network construction, we used the 12,253 genes which passed the quality control measures as described above. Again, we selected the most varying genes by using the variance threshold of S.D. > 0.25, resulting in 8,745 genes for network construction. The adjacency matrix was constructed in both sub-networks, and was created by calculating the Pearson’s correlations and raising them to the power β = 9, based on the scale-free topology assumption. The connectivity of each gene was calculated in both the lean and obese sub-network, as the sum of connection strengths of a particular gene with all other genes.

The differential connectivity (k_diff) was calculated by subtracting the connectivity of the genes in the lean sub-network from the connectivity in the obese sub-network. This resulted in a normal distribution of values between -1 and 1. When the k_diff was positive, it meant that the genes were highly connected in the obese sub-network than in the lean sub-network, while a negative k_diff meant that the genes were highly connected in the lean sub-network than in the obese sub-network. Furthermore, we performed a standard *t*-test, comparing the gene expression of the obese vs. the lean animals, to assign the difference in expression between the two sub-networks. Genes were labeled as differential connected when the absolute k_diff was above 0.6, resulting in the detection of genes which were hubgenes in one sub-network, but not in the other sub-network. Differentially connected genes were selected for functional annotation when their absolute *t*-test statistic was above 1.96 indicating that the genes are differentially expressed between the obese and lean animals. Associated genes were detected again using BioMart [107].

### Detection of regulator genes

**Figure 6:**
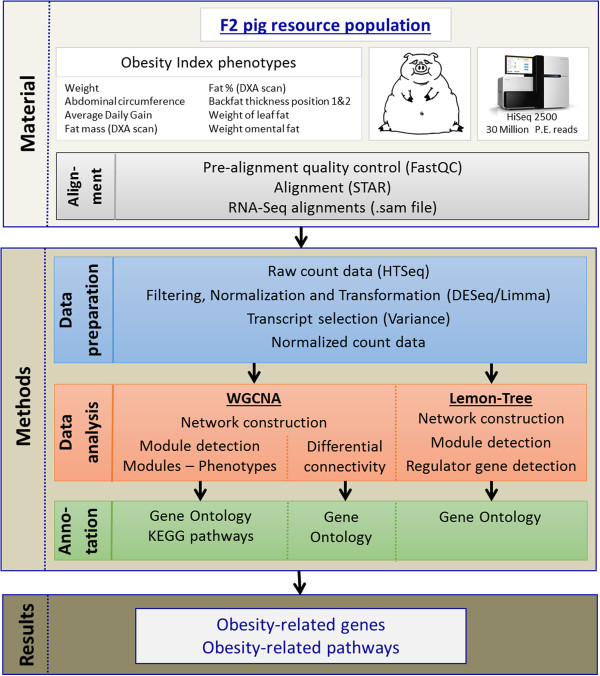
Workflow of the RNA-Seq data analysis.

To detect regulator genes of the modules, we used the Lemon-Tree software suite, available at https://code.google.com/p/lemon-tree/. Lemon-Tree is able to detect regulatory modules from gene expression data using probabilistic graphical models [109]. We used the voom() normalized expression data of the 12,253 genes which passed QC, and further reduced the dataset by selecting genes with a standard deviation above 0.5. Afterwards, the 3,101 resulting genes were centered and scaled, resulting in a mean of 0 and standard deviation of 1. Using Lemon-Tree, expression data is clustered based on the Gibbs sampler method [110]. To identify reliable clusters the clustering algorithm was run 10 times, and afterwards clusters are integrated to generate a single robust clustering solution (tight clustering). Samples were grouped (hierarchical tree) based on a similar mean and standard deviation. To identify candidate regulator genes we created a set of potential regulators consisting of transcription factors and signal transducers, using the GO categories ‘transcription factor activity’ and ‘signal transducer activity, resulting in 1104 genes. Those potential regulators were assigned to nodes in the hierarchical tree by logistic regression, and probabilistic scores were assigned to those regulators. This results in a high probabilistic score in case there is a different expression pattern on each side of the particular tree node. After generation of multiple statistically equal partitions of conditions for each cluster of genes, it uses an ensemble approach to sum the strength with which a regulator participates in each regulatory tree. The overall statistical confidence is calculated and used for prioritizing regulators, represented by the global probabilistic score (Prob. score). We calculated the significance of those probabilistic scores by comparing the assigned regulators with randomly assigned regulators, using a *t*-test comparing their means. Mathematical details of the Lemon-Tree algorithms can be found in Joshi et al. [16].The complete workflow used in this study is visualized in Figure 6.

### Availability of supporting data

The data discussed in this publication have been deposited in NCBI's Gene Expression Omnibus [111] and are accessible through GEO Series accession number GSE61271 (http://www.ncbi.nlm.nih.gov/geo/query/acc.cgi?acc=GSE61271) [98].

## Competing interests

The authors declare that they have no competing interests.

## Authors’ contributions

HNK was the overall project leader who conceived and conducted this study and supervised LJAK in the genetics, bioinformatics and systems biology analyses. MF collected and provided the phenotypic measurements and adipose tissue for RNA Sequencing. SC performed the RNA isolation, and quality control of RNA samples before RNA Sequencing. LJAK analyzed all the data and wrote the first draft of the manuscript. Preliminary analytical work was carried out at the University of Groningen Medical Centre where DVZ helped LJAK by creating the RNA Sequencing alignment pipeline, under the supervision of LF. All authors wrote, read, and approved the final version of the manuscript.

## Pre-publication history

The pre-publication history for this paper can be accessed here:

http://www.biomedcentral.com/1755-8794/7/57/prepub

## Supplementary Material

Additional file 1 All genes present in the selected WGCNA modules (Black, Blue, Brown, Lightyellow, Greenyellow) with the ensemble gene ID, position and associated gene names (using BioMart); genes which were not assigned to a gene name are not presented. Click here for file
